# Role of Homologous Recombination Repair (HRR) Genes in Uterine Leiomyosarcomas: A Retrospective Analysis

**DOI:** 10.3390/cancers14081934

**Published:** 2022-04-12

**Authors:** Francesca Ciccarone, Matteo Bruno, Elisa De Paolis, Alessia Piermattei, Maria De Bonis, Domenica Lorusso, Gian Franco Zannoni, Nicola Normanno, Angelo Minucci, Giovanni Scambia, Gabriella Ferrandina

**Affiliations:** 1 UOC Ginecologia Oncologica, Dipartimento per la salute della Donna e del Bambino e della Salute Pubblica, Fondazione Policlinico Universitario A. Gemelli, IRCCS, 00168 Rome, Italy; francesca.ciccarone@policlinicogemelli.it (F.C.); brunomatteo2@gmail.com (M.B.); kettalorusso@libero.it (D.L.); gabriella.ferrandina@gmail.com (G.F.); 2 Molecular and Genomic Diagnostics Unit, Fondazione Policlinico Universitario Agostino Gemelli IRCCS, 00168 Rome, Italy; elisa.depaolis@policlinicogemelli.it (E.D.P.); maria.debonis@tim.it (M.D.B.); angelo.minucci@policlinicogemelli.it (A.M.); 3 Gynecopathology and Breast Pathology Unit, Department of Woman and Child’s Health and Public Health & Sciences, Fondazione Policlinico Universitario Agostino Gemelli IRCCS, 00168 Rome, Italy; alessia.piermattei@policlinicogemelli.it (A.P.); gianfranco.zannoni@unicatt.it (G.F.Z.); 4 Istituto di Ginecologia e Ostetricia, Università Cattolica del Sacro Cuore, 00168 Rome, Italy; 5 Cell Biology and Biotherapy Unit, Istituto Nazionale Tumori “Fondazione G. Pascale”-IRCCS, 80131 Naples, Italy; nicnorm@yahoo.com

**Keywords:** uterine sarcomas, molecular characterization, homologous recombination deficiency, clinical outcomes

## Abstract

**Simple Summary:**

A more in-depth molecular characterization of uterine leiomyosarcomas (uLMS), a rare disease characterized with dismal prognosis, could provide data suitable for the identification of potential target-based drugs. We aimed to define frequencies of gene alterations in uLMS, especially regarding the somatic mutations of *BRCA* and HRR gene alterations, and identify the impact of these molecular alterations on clinical outcomes. This retrospective analysis of the mutational profile of uLMS showed that the most frequent alterations involved the *TP53* gene, and that patients with *TP53* alterations experienced a worse prognosis compared to patients with wild-type *TP53* genes. Conversely, patient clinical outcomes were similar within patients with *BRCA-* and HRR-related genes versus non-HRR-related genes. However, although the frequency of patients with *BRCA-* and HRR-related alterations and mutations was relatively small, this setting could deserve an investigation into drug actionability, and potentially benefit from PARP inhibitors.

**Abstract:**

Uterine leiomyosarcomas (uLMS) is a very rare disease, and patients experience a dismal prognosis even when treated with chemotherapy. Therefore, a more in-depth molecular characterization of this disease could provide suitable data for the identification of potential target-based drugs. This retrospective, single institutional study aimed to define the frequencies of gene alterations in uLMS, especially regarding the somatic mutations of *BRCA* and Homologous Recombination Repair (HRR) genes, and the impact of molecular alterations on clinical outcomes. The 16-genes Next-Generation Sequencing (NGS) panel, Homologous Recombination Solution TM (HRS, Sophia Genetics, Saint Sulpice, Switzerland), was used for the molecular evaluation of samples. The majority of patients (66/105, 63%) carried at least one sequence alteration, with a prevalence of *TP53* involvement followed by *RAD51B*, *BRCA1/2*, and *FANCL*. Patients with *TP53* gene alterations experienced a significantly worse prognosis for progression free survival (PFS) and overall survival (OS) versus wild-type patients. Given the number of patients with the *BRCA1/2* mutation (N = 12), we included them in the HRR patient group; there was no difference in clinical outcomes with HRR versus non-HRR. The Cox’s multivariate analysis showed that stage and *TP53* gene alterations resulted in a significantly worse OS. The integration of gene networking data, such as tumor mutation burdens and cancer driver gene identification, could show a clearer discrimination of gene distribution patterns, and lead to the implementation of therapeutic targets.

## 1. Introduction

Uterine sarcomas are very rare malignancies, accounting for 3–7% of uterine malignancies. Uterine leiomyosarcomas (uLMSs) represent around 60–70% of cases, with an incidence of 0.5–0.8 new diagnoses/100,000 women/per year [1]. A spectrum of uLMS subtypes have been recognized, including spindle cell morphology, which is the most frequently documented, followed by the myxoid and epithelioid variants [2,3]. Even though almost 60% of uLMSs are diagnosed at stage I, relapse occurs in 30–60% of cases, with distant disease sites accounting for about 70% of recurrences [4]. The 5-year overall survival is around 75% in stage I, 60% in stage II, and 30–45% in stage III-IV [5]. According to the NCCN, ESMO and Italian guidelines, adjuvant chemotherapy is not recommended in early-stage uLMS [6,7,8]. Conversely, chemotherapy has been demonstrated to improve clinical outcomes in advanced/recurrent patients, even though the percentage of response is only around 10 to 50% [9]. Several data from phase III randomized studies have confirmed the efficacy of doxorubicin as single agent or in combination, followed by trabectedin, or gemcitabine/docetaxel [10,11,12]. However, the current options for target-based drugs for this disease are limited only to oral pazopanib [13].

In this context, a more in-depth molecular characterization of this disease could hopefully provide a biologic platform for the investigation of potential target-based drugs.

Several lines of evidence have highlighted the peculiarity of uLMS compared to extra-uterine sarcomas [14,15,16,17,18,19]. In recent years, much attention has been focused on the role of *BRCA* genes and homologous recombination deficiency (HRD) in soft tissue sarcomas [20,21,22,23,24]. In particular, the comprehensive and integrated genomic characterization of adult soft tissue sarcomas has demonstrated that uLMSs are characterized by a higher level of hypomethylated estrogen receptor-dependent target genes, DNA damage response scores, and genomic instability compared to extra-uterine sarcomas [18]. Moreover, a large comprehensive series registered a higher prevalence of *BRCA2* and Homologus Recombination Repair (HRR) gene mutations in uLMS compared to extra-uterine sarcomas [22].

More recent studies have investigated the potential association of genomic alterations with clinical outcomes [19,23,24,25,26,27]. In this context, the most frequent alterations were loss-of-function mutations in *TP53* (56%), *RB1* (51%), and *ATRX* (31%), whereas homozygous deletions of *BRCA2* genes were present in only 5% of patients [23].

The present study was aimed at defining: (i) the frequency of *BRCA* and HRR-related gene mutations in uLMS, and (ii) the impact of genomic alterations on patient clinical outcomes.

## 2. Material and Methods

### 2.1. Patient Selection

This was an observational, single institutional, retrospective study aimed at defining: (i) the frequency of tumor suppressor alterations in uLMS, and (ii) the impact of molecular alterations on patient clinical outcomes.

The primary endpoint was to evaluate the frequency of the somatic mutations of *BRCA* and HRR-related genes in uLMS. The secondary endpoint was to identify the association of somatic mutations of *BRCA* and HRR-related genes with clinical outcomes, i.e., progression-free survival (PFS) and overall survival (OS).

After obtaining the Institutional Review Board’s (ID 3538) approval, we collected 112 uterine LMS tumor samples from 105 patients. All cases of uterine sarcomas included were taken from the Fondazione Policlinico Universitario Agostino Gemelli IRCCS database, from December 2008 to July 2020. We retrieved the histological and clinical data of patients from clinical charts and from the “Uterine Sarcomas” database of Fondazione Policlinico Universitario Agostino Gemelli, IRCCS. All patients had already provided written informed consent to collect and analyze their data for scientific purposes. Data analysis was performed after the anonymization of patients.

Inclusion criteria were: pathologically confirmed diagnosis of primary and recurrent uLMS, availability of formalin-fixed and paraffin-embedded (FFPE) tissue at the diagnosis and, availability of clinical information (baseline information, surgery, adjuvant therapy, and at least 1 year follow-up).

Exclusion criteria were: metastatic cancer of the uterus, clinical information not available or uncompleted, and any biological therapy in the past 5 years.

The following data were retrieved from medical records: tumor histology and International Federation of Gynecology and Obstetrics (FIGO) stage at the time of initial diagnosis, data related to surgery and adjuvant treatments (chemotherapy or radiotherapy), and histopathology reports. As far as histopathological/immunohistochemical characteristics are concerned, we planned to collect data on tumor cellularity, tumor necrosis, estrogen and progesterone receptors, and Ki67. PFS was calculated from the date of diagnosis to the documentation of radiological disease progression or the date of the last follow-up. OS was calculated from the date of diagnosis to the death of disease or the date of the last follow-up.

### 2.2. Tissue Samples, DNA Extraction and Quality Evaluation

All hematoxylin and eosin (HE) staining data related to the diagnosis of leiomyosarcoma were collected and evaluated by a dedicated gyneco-pathologist to determine the percentages of tumor cells (%TCs) and tumor necrosis (%TN) in the macrodissected areas [28]. FFPE tissue blocks, representative of the tumor lesions, were collected and cut into 10 μm thick slides. DNA was extracted from the 10 μm thick unstained FFPE slide using the MagCore^®^ Genomic DNA FFPE One-Step kit (RBC Bioscience, New Taipei City, Taiwan) on the automated platform MagCore^®^ HF16Plus (Diatech Lab Line, Jesi, Italy), following the manufacturer’s instructions. The quantitation of the extracted DNA was performed using Qubit dsDNA HS fluorimetric assays (Life Technologies, Gaithersburg, MD, USA). Finally, the qualitative evaluation of the DNA integrity was performed using the DNA Fragmentation Quantification Assay (EntroGen Inc., Los Angeles, CA, USA) on the LightCycler^®^ 480 Real-time system (Roche Diagnostics, Basel, Switzerland). Only tissue samples with at least 50% of amplifiable DNA ≥ 150 bp were analyzed.

### 2.3. Multi-Gene Panel Test and Next-Generation Sequencing

The 16-genes Next-Generation Sequencing (NGS) panel, Homologous Recombination Solution TM (HRS, Sophia Genetics, Saint Sulpice, Switzerland), was used for the target molecular evaluation of samples. The multi-gene panel covered the coding regions and splicing junctions of the following 16 genes involved in the homologous recombination pathway: *ATM* (NM_000051.3), *BARD1* (NM_000465.3), *BRCA1* (NM_007294.3), *BRCA2* (NM_000059.3), *BRIP1* (NM_032043.2), *CDK12* (NM_015083.2), *CHEK1* (NM_001114121.2), *CHEK2* (NM_001005735.1), *FANCL* (NM_001114636.1), *PALB2* (NM_024675.3), *PPP2R2A* (NM_002717.3), *RAD51B* (NM_02877.5), *RAD51C* (NM_058216.2), *RAD51D* (NM_002878.3), *RAD54L* (NM_001142548.1), and *TP53* (NM_000546.5). For each analyzed sample, sequencing libraries were prepared, starting from 100 ng of DNA using the Kapa HyperPlus library preparation kit (Roche Diagnostics, Basel, Switzerland), according to the manufacturer’s protocol. DNA fragments were generated using an enzymatic fragmentation step. The three subsequent enzymatic steps, end-repair, A-tailing, and ligation to Illumina adapters, were performed in order to produce NGS libraries. A capture-based target enrichment was carried out on the pooled libraries. The quantitation of the final pool of libraries was performed using Qubit dsDNA HS fluorimetric assays (Life Technologies, Gaithersburg, MD, USA). Quality control of fragment size was assessed using DNA ScreenTape analysis (4200 TapeStation system, Agilent Technologies, Palo Alto, CA, USA). A total of 3 NGS runs with 48 libraries/run at the final concentration of 1.3 pM were performed using Illumina Mid Output kit v2 (300 cycles). NGS protocol was then performed in paired-ends reads mode with FASTQ only analysis workflow on the Illumina NextSeq550DX^®^ NGS platform (Illumina, San Diego, CA, USA).

### 2.4. Mutational Analysis, CNV Prediction and Variant Classification

Data analysis was performed in order to detect Single Nucleotide Variants (SNVs), insertions/deletions (indels), and Copy Number Alterations (CNAs). Sequencing FASTQ data were analyzed by the Sophia DDM^®^ platform (Sophia Genetics, Saint Sulpice, Switzerland). The Minimum Allele Frequency (MAF) threshold was fixed as equal to 5%. Variants with a mean coverage below 300× were excluded from the analysis. The bioinformatic prediction of CNAs was performed by analyzing the coverage levels of the target regions across samples. For each sample, the algorithm automatically selects a set of reference samples from the same run, based on the similarity of coverage patterns. The coverage is normalized by sample and by the target region, and CNA detection is performed using a hidden Markov model algorithm. As a result, the most likely copy number for each target region is determined. If, after attributing the copy number values, the residual noise remains high within a particular gene in a particular sample, this gene and/or the sample is marked as rejected. The CNA module detects partial CNAs, with the resolution of a single exon, but not the whole gene copy number alteration. The detection, annotation and pre-classification of each genomic variant were performed according to the American College of Medical Genetics and Genomics (ACMG) guidelines. The final variant classification was obtained by querying online databases including dbSNP (https://www.ncbi.nlm.nih.gov/snp/; accessed on 6 April 2022), 1000 Genomes (http://www.internationalgenome.org/; accessed on 6 April 2022), ClinVar (http://www.ncbi.nlm.nih.gov/clinvar/; accessed on 6 April 2022), LOVD (https://www.lovd.nl/; accessed on 6 April 2022), ENIGMA (https://enigmaconsortium.org/; accessed on 6 April 2022), IARC *TP53* Database (http://p53.iarc.fr/; accessed on 6 April 2022), OncoKb (https://www.oncokb.org/; accessed on 6 April 2022), Cancer Genomic Interpreter (https://www.cancergenomeinterpreter.org/home; accessed on 6 April 2022), VARSOME (https://varsome.com/; accessed on 6 April 2022), and COSMIC (https://cancer.sanger.ac.uk/cosmic; accessed on 6 April 2022). Novel variants resulting in a shift of the reading frame and/or in a premature stop of the protein translations were classified as likely pathogenic, according to the ACGM guidelines. All genomic alterations accounted in ATM, *BARD1*, *BRCA1*, *BRCA2*, BRIP1, CHEK2, PALB2, RAD51C, RAD51D and *TP53* genes were re-evaluated and confirmed with alternative NGS protocols. Briefly, the amplicon-based NGS kits HBOC Devyser and *BRCA* Devyser (Devyser, Hägersten, Sweden) were used for library preparation, according to the manufacturer’s instructions. Sequencing reactions were carried out on the Illumina MiSeq System (Illumina, San Diego, CA, USA) and CE-IVD Amplicon Suite Software (SmartSeq, Novara, Italy) was used for variant calling and CNV evaluation.

### 2.5. Statistical Analysis

PFS was calculated as the time elapsed from the date of diagnosis to the date of first recurrence or last follow-up. OS was calculated as the time from the date of diagnosis to the date of death of disease or the date of last follow-up. Medians and life tables were computed using the product limit estimated by the Kaplan–Meier method, and the log-rank test was used to assess the statistical significance [29,30]. The Cox regression model was used to perform the multivariate analysis of the prognostic factors [31]. All statistical tests were performed using the Statistical product and Service Solutions software (SPSS, version 22.0; BM Corp., Armonk, NY, USA). Statistical tests were two-sided, and differences were considered significant at the level of *p* value < 0.05.

## 3. Results

### 3.1. Patient Characteristics

**Figure 1:**
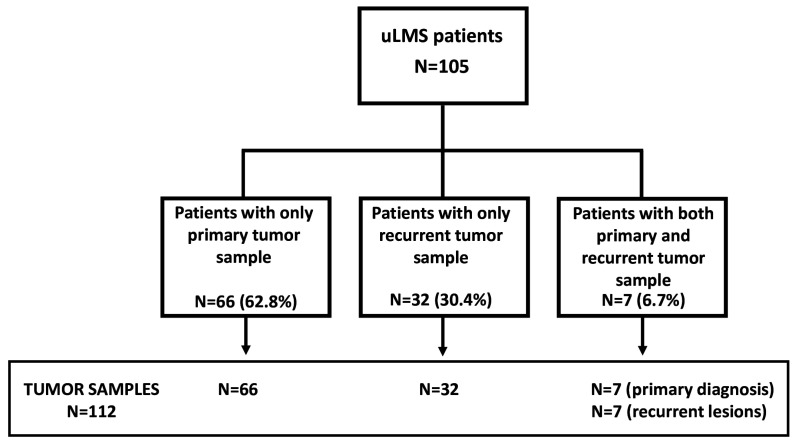
Flow chart of patients and tumor tissue samples.

As shown in Figure 1, one hundred and five patients were included in this study. Table 1 summarizes the patient’s features: sixty-seven patients (63.8%) reported a family history of oncologic disease. The median age was 52 years (range: 25–94); early stage was shown in 81 patients (77.1%), and spindle histology was documented in 73 patients (69.5%), followed by mixed (12.4%), epithelioid (9.5%), and mixoid (8.6%) ones. At diagnosis, the vast majority of patients (69.5%) were managed by primary cytoreduction, while neoadjuvant chemotherapy followed by interval debulking surgery was received by 30.5% of patients. Hysterectomy and salpingo-oophorectomy were most frequently carried out at the time of cytoreduction, and laparotomy was the most common surgical approach; additional surgical procedures on disease sites were mainly represented by pelvic peritonectomy followed by omentectomy, bowel resection, bladder or pulmonary resection at the time of recurrence. Salvage and adjuvant chemotherapy were administered to 89 patients; radiotherapy was adopted in early-stage patients with close tumor margins for local disease control (N = 12), and in advanced patients for the palliation of symptoms (N = 7).

### 3.2. Tumor Sample Features

Of the 105 patients, we obtained tumor samples from the initial diagnosis in 66 patients, at the initial diagnosis and recurrence in 7 patients, and at the time of recurrence in 32 patients, leading to a total of 112 lesions (Figure 1). Table 2 reports the histological and immunohistochemical analysis of the lesions: most samples presented >80% tumor cellularity (N = 70, 62.7%), or within 61–80% (N = 29, 25.8%), while tumor necrosis was <20% in most of the samples (N = 91, 81.2%), or within 21–40% (N = 14, 12.5%). The median of estrogen receptor expression was 21% (range: 0–100), and 28% (range: 0–90) in primary and recurrence samples, respectively; the median of progesterone receptor expression was 15% (range: 0–100) in primary samples and 18% (range: 0–100) in recurrent lesions. There was no difference across ER and PR between primary and recurrent tissue samples. The median expression of Ki67 was 48% (range: 10–90).

### 3.3. Clinical Outcomes

As of December 2020, the median follow-up was 28 months (range: 1–156). Of the 105 patients, relapse of disease was documented in 72 patients (68.5%), 51 patients experienced recurrent abdominal disease, and 21 patients showed extra-abdominal disease (lung, bone or subcutaneous metastases). The 3-year PFS rate was 28.1%, and median PFS was 16 months (range: 1–111); the 3-year OS rate was 56.8%, and median OS was 33 months (range: 4–156) (Figure S1A).

### 3.4. Genomic Characterization

**Figure 2:**
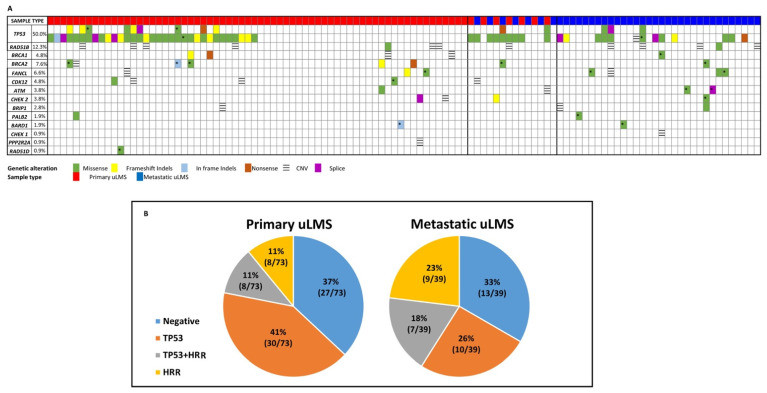
Molecular profile of uLMS. (**A**) Genomic alterations found in enrolled patients. For *TP53*, double rows represent the co-occurrence of two unique variants in the same uLMS case. (**B**) Distribution of frequencies of alterations in *TP53* gene (orange), HRD-related genes (gray), and both *TP53* and HRD-related genes (gold) on primary and metastatic lesions. Wild-type genes are included in the blue panel. “*” Variants of Uncertain Significance (VUSs).

The overall distribution of genetic alterations found in the cohort is reported in Figure 2A. The majority of patients (66/105, 63%) carried at least one sequence alteration. Consistent with the literature, *TP53* was found to be the most frequently mutated gene, with 50% (52/105) of the patients having at least one alteration. Additionally, 39% of patients (41/105) harbored HRR-related gene alterations. Among these, *BRCA1/2* genes were the most commonly involved genes (12/105, 11.4%), with *BRCA2* alterations being the most prevalent, followed by the *FANCL* gene (7/105, 6.6%). Among the patients harboring genetic alterations, the most common molecular background was the presence of only one variant per gene (57%), particularly common for the *TP53* gene (39%), or an HRR-related gene (18%). The NGS pipeline used in this study allowed for the prediction of CNAs in the genes included in the panel. Twenty-five percent of patients carried a CNA, generally presenting as multiple events together with SNVs in the same patient. Overall, a widespread involvement of the analyzed genes was observed, with no difference between the frequency of deletions or amplifications. Contrary to what was observed in the sequence variants evaluation, CNAs in *TP53* and *BRCA* genes were found to be rare events, with only one *TP53* deletion and one *BRCA2* deletion. Instead, a high prevalence of *RAD51B* involvement was observed (Figure S2).

A differentiated molecular analysis was performed in the groups of primary tumors and metastatic disease (Figure 2B). *TP53* was found to be the most involved gene in both groups and a higher percentage of molecular defects in HRR-related genes was observed in the metastatic disease. We observed a higher frequency of *BRCA2* alterations in the primary group compared to the metastasis, with five and two variants, respectively. Instead, a comparable distribution among the group was observed for the remaining genes.

In the CNAs evaluation, no significantly different frequencies were observed between primary (16%) and metastatic disease (13%). In addition, 61% of the alterations identified in the primary group were missense variants, followed by indels (27%), splice-site (7.5%) and nonsense (4.5%). Additionally, in the metastatic group, the missense variants were the most frequent (76%), and a similar proportion of the nonsense variants was observed (6%). Unlike the primary group, we observed a higher frequency of splice-site variants (12%) and a lower frequency of indels (6%) in the metastatic lesions.

**Figure 3:**
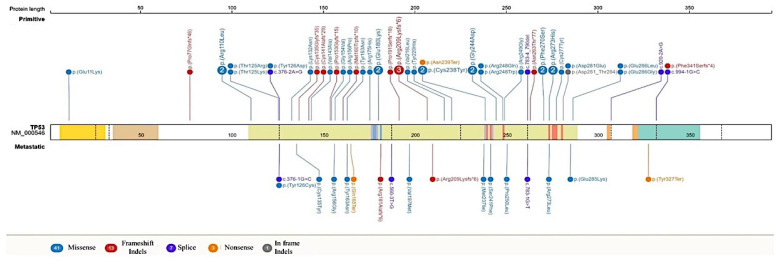
Distribution of *TP53* variants in the context of a protein structure. The figure shows the linear maps of *TP53* and the location of the genetic variants.

The *TP53* variants identified were scattered throughout the genes, with several hot-spot codons: 125, 126, 180, 209, 238, 244, 270, 273, 281, 286. Interestingly, the oncogenic *TP53* variant c.626_627del (p.Arg209Lysfs*6) was identified as a recurrent alteration in our cohort (Figure 3).

Finally, we performed a molecular evaluation of the seven patients for which both primary tumor and matched metastasis samples were available. In this group, six patients carried out at least one genetic alteration. Despite the low number of subjects, we observed that all the patients showed a different molecular background in the metastatic samples when compared to the matched primary tumors. Particularly, four out of six patients showed an additional molecular impairment; this was mainly mediated by *TP53* gene status via the occurrence of a new somatic *TP53* variant in the metastatic disease sample (two cases), or via an increased VAF% in the primary tumor sample (two cases).

Notably, one patient acquired a *BRCA2* variant together with a *TP53* alteration in the metastatic disease sample. In the remaining two metastatic disease cases, we observed the absence of the *TP53* variant previously identified in the primary matched tumors. In relation to this, we performed a thorough data assessment in terms of: (i) tumor biopsy and tumor cell content; (ii) variant calling quality of both primary and matched metastatic disease samples; (iii) a re-evaluation of low confidence or unfiltered variants; and (iiii) the absence of CNAs in *TP53* genes. No specific CNA involvement was identified in the group of primary and matched metastatic disease samples, probably due to the low number of cases available.

Details regarding sequence variants and CNA events found across the entire cohort of the study are available in Table S1.

### 3.5. Survival Analysis Based on Molecular Characterization

Survival analysis was performed only on patients (*n* = 73) with tumor sample tissue at primary diagnosis: the 3-year PFS was 36.1%, and median PFS was 18 months (1–129). The 3-year OS was 50.4%, and the median OS was 33 months (range: 4–156) (Figure S1B).

We performed a survival analysis of patients with the most frequent genomic alterations, i.e., *TP53*, HRR-related genes, and CNA.

**Figure 4:**
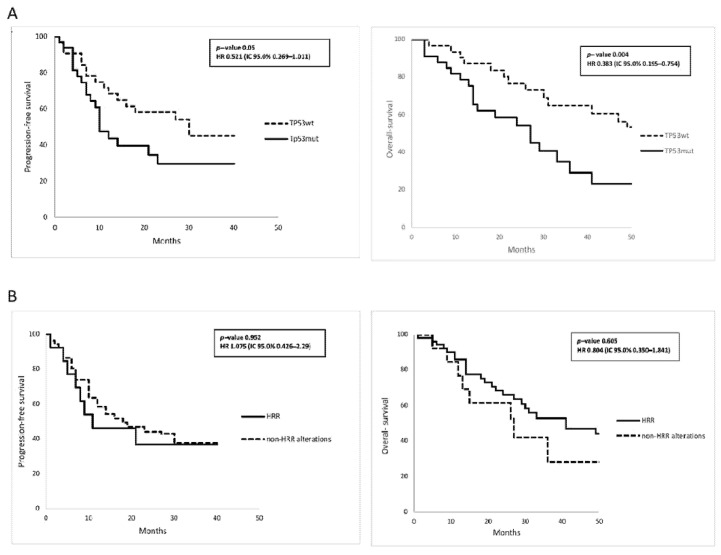
PFS and OS according to patients with: (**A**) wild-type *TP53* versus mutated *TP53*; (**B**) HRR versus non-HRR alterations.

As shown in Figure 4A, the 3-year PFS was 29.7% in patients with *TP53* mutations versus 45.0% in patients with wild-type *TP53* (*p* value = 0.050). Moreover, we reported that the 3-year OS was 29.1% in patients with *TP53* mutations versus 65.0% in wild-type *TP53* (*p* value = 0.004).

In Figure 4B, the PFS and OS analyses showed no significant difference between patients with HRR-related versus non-HRR-related alterations: the 3-year PFS was 36.7% in patients with HRR, versus 36.9% in patients with non-HRR alterations (*p* value = 0.938); the 3-year OS was 52.9% in patients with HRR, versus 28.1% in patients with non-HRR alterations (*p* value = 0.605).

As far as CNA status is concerned, there was no difference in clinical outcomes in terms of the presence or absence of CNA mutational status: the 3-year PFS was 33.3% in patients presenting with CNA versus 42.8% in patients without CNA (*p* value= 0.938); the 3-year OS was 52.9% in patients presenting with CNA versus 28.1% in patients without CNA (*p* value = 0.790) (Figure S3).

We also analyzed the most prevalent genomic alterations (*TP53*, HRR, CNA) and stages of disease by multivariate Cox analysis (Table 3). Stage of disease had a statistically significant impact on PFS (HR:0.338; CI95%:0.161–0.709, *p* value = 0.004), and a borderline significant impact on OS (HR:0.474; CI95%:0.224–1.006, *p* value = 0.052). Among genomic alterations, *TP53* showed a borderline significant impact on PFS (HR:0.510; CI95%:0.246–1.056, *p* value = 0.070), and a statistically significant impact on OS (HR:0.312; CI95%:0.145–0.670, *p* value = 0.003).

## 4. Discussion

Our study analyzed the prevalence of somatic genomic alterations in HRR-related genes in a single institutional series of uLMS, and the impact of these genomic alterations on patient clinical outcomes.

To date, many studies about the uLMS molecular landscape have been published [18,19,20,21,22,23,24,25]; the vast majority of these studies have analyzed wide cohorts of patients with several types of soft tissue LMS, as well as uLMS [18,19,20,21,22]. Indeed, in recent years, the peculiarity of uLMS compared to extra-uterine sarcomas has been well defined based on the TCGA study [14,15,16,17,18,19]: uLMS are characterized by a higher level of hypomethylated estrogen receptor-dependent target genes, as well as DNA damage response scores, and genomic instability compared to extra-pelvic sarcomas [18]. uLMS are also characterized by a complex karyotype with low mutational burden, and recurrent alterations in a few key tumor suppressor genes, such as *TP53*, *RB1*, *ATRX*, and *PTEN*, and are thus associated with widespread DNA CNAs [18,19,20,24,25]. Moreover, Rosenbaum et al. [24] reported a percentage of 18.2% of genomic HRD alterations (including *BRCA1/2* genes) in uLMS, versus 10.0% in soft tissue sarcomas.

In our study, the most frequent alterations were registered in the *TP53* gene (50%), a finding that matched well with previous studies [23,25]. The *TP53* gene is a tumor suppressor engaged in cellular death, cellular reprogramming, the remodeling of chromatin, and drug resistance [32]; therefore, alterations to the *TP53* gene lead to an escalation of DNA mutagenesis by the inactivation of other tumor suppressor genes, such as the RB1 gene, which controls cell cycle and genomic stability, and the *PTEN* gene, which regulates PI3K/AKT/mTOR signaling. There, this gene plays a relevant role in terms of cell growth and apoptosis [32]. Among the *TP53* variants detected in our cohort, *c.626_627del* (p.Arg209Lysfs*6) was found to be recurrent alteration (*n* = 3 primary and *n* = 1 metastatic disease), mainly with a high VAF%. This variant was previously confirmed as a somatic variant in patients affected by breast carcinoma of different histological subtypes, and in a series of other cancer types (e.g., colorectal, brain, lung, and ovarian), according to the IARC *TP53* database and the COSMIC database. Moreover, it is not present in the main population databases (1000 Genomes), and it was reported as a germline variant in individuals affected with embryonal rhabdomyosarcoma [33], in a family with very-early-onset breast cancer and osteosarcoma [34], and in an ovarian cancer case [35]. Interestingly, two metastatic disease cases showed the absence of the somatic *TP53* variants previously identified in their primary matched tumors. Identifying the reasons for this is challenging. Historically, tumorigenesis has been considered an evolutionary process related to the accumulation of somatic variants [36]. However, tumor heterogeneity, genetic clonality, and divergence in relation to metastatic tumor cell dynamics are still controversial topics that have not been fully elucidated. *TP53* alterations give selective advantage to cancerous cells, and they are considered oncogenic drivers of events. However, single genetic changes are unlikely sufficient for metastasis, and alternative models of cancer progression have been proposed [37]. Furthermore, we cannot rule out the impact of tumor cell response to the therapies administrated between primary tumor and metastatic disease diagnosis, as previously demonstrated [38].

Finally, somatic CNAs account for more than 80% of all copy number changes detected in endometrial carcinomas and uterine sarcomas [39]; these alterations comprise inversions, translocations, amplifications, and deletions of DNA sequences, ranging from sub-microscopic events to complete chromosomal aneuploidies. These alterations are strongly associated with developmental disorders and human tumorigenesis [39]. In this context, we would underline that the bioinformatics pipeline adopted in this study allowed CNA prediction up to the deepest possible single-exon alterations. Obtaining CNA analysis from FFPE-derived NGS data is not easy, and it generally relies on the whole gene CNA evaluation. In our opinion, our approach is useful for a gene-level investigation, and the study has translational relevance. For example, CNAs involving single or few *BRCA1/2* exons are considered relevant findings in Hereditary Breast and Ovarian Cancer (HBOC) syndrome for PARP inhibitor administration [40].

Concerning genes related to HRD, we confirmed that the percentage of *BRCA 1/2* gene alterations was 11.4%, with a prevalence of *BRCA2* (7.6%), as previously reported [24,25]. Additionally, in the present paper, we underline the involvement of the *BRCA1* gene in 4.8% of the enrolled patients as an additional finding. Moreover, we detected *RAD51B* in 12.3%, and *FANCL* in 6.6%, similar to other previous studies [22,25]. HRR genes play key roles in restoring the original DNA sequences in cases of double-strand break damages [41,42]; therefore, the loss of function in HRR proteins leads to the impairment of this machinery, i.e., homologous recombination deficiency (HRD), due to the alterations of key genes such as *BRCA1/2*, *RAD51C*, *RAD51D*, and *PALB2* [43].

As far as clinical outcomes are concerned, we showed that patients with mutated *TP53* genes experienced a worse prognosis both for PFS and OS; these results differ to the series published by Astolfi et al. [25], who did not show any significant correlation between the mutational statuses of *TP53*, *RB1*, *ATRX*, or *PTEN*. This could likely be ascribed to some issues, such as the relatively large rate (62%) of patients with advanced stage disease, and different therapeutic managements across three databases and three institutions [25]. Therefore, we tried to understand whether the use of the Cox’s multivariate analysis could distinguish the alterations of genes with potential impact on clinical outcomes; indeed, besides the stage, only the presence of *TP53* gene alterations resulted in a statistically significant worse OS. Since the number of patients with the *BRCA1/2* mutation was only 12, we included them with patients with other HRR gene alterations; however, there was no difference in patient clinical outcomes with HRR-related versus non-HRR-related genes. In this context, Rosenbaum et al. [24] reported that patients with HRD alterations experienced a statistically significantly shorter PFS compared to wild-type patients, while there was no difference in OS between patients in the homologous recombination pathway-altered group versus the wild-type group [24]. Unfortunately, these data are difficult to explain, since the sample was heterogenous, thus including male and female patients, several histotypes of LMS and uLMS, and even different samples of PFS (N = 110) and OS (N = 211).

Despite efforts to characterize molecular targets which might lead to novel therapeutic scenarios, at the moment, uterine sarcomas are still managed by standard chemotherapy, with oral pazopanib as the only target-based drug [13]. However, within recent years, some lines of evidence have collected data on HRD in uLMS, with a prevalence of around 6.5% to 23.0% [22,23,24,44,45]. Moreover, two studies have reported the use of olaparib in nine patients with *BRCA2* genomic alterations [22,23]; overall, three patients had stable disease, five patients achieved disease regression, and one obtained a complete radiographic response. Moreover, some instances of preclinical data have reported that LMS patient-derived xenografts (PDXs) (Ley16) associated with the gain of the functions PTEN, c-MYC, and/or HRD signatures, when treated by a PARP-inhibitor (Olaparib), resulted in a slower rate of tumor proliferation compared to the control [26]. Therefore, since genetic and epigenetic abnormalities leading to HRD in human malignancies are actionable thanks to the recent development and exploitation of PARP-inhibitors, novel therapeutic scenarios could be expected to tackle this disease.

## 5. Conclusions

In summary, this retrospective analysis of uLMS mutational profiles shows that the most frequent alterations involved the *TP53* gene, and that patients with *TP53* alterations experienced a worse prognosis compared to patients with wild-type *TP53* genes. On the other hand, patient clinical outcomes were superimposable within patients with *BRCA* and HRR-related genes compared non-HRR-related genes. However, although the frequency of patients with *BRCA-* and HRR-related mutations was relatively small, this setting could warrant an investigation into drug actionability, and potentially benefit from PARP inhibitors. The integration of gene networking data with further variables, such as tumor mutation burdens and cancer driver gene identification, could show a clearer discrimination of gene distribution patterns with the implementation of specific markers and therapeutic targets.

## Figures and Tables

**Table 1:** Baseline characteristics of uLMS patients.

Patient Characteristics	uLMS Group (N = 105)
Age, yrs	
median (range)	52 (25–94)
Menopause	
Yes	53 (50.4%)
No	48 (46.1%)
*n.a.*	4 (3.5%)
Familiarity cancer risk	
Yes	67 (63.8%)
No	34 (32.8%)
*n.a.*	4 (3.4%)
FIGO stage	
I	71 (67.6%)
II	10 (9.5%)
III	9 (8.5%)
IV	14 (13.4%)
*n.a.*	1 (1.0%)
Histotype	
Spindle	73 (69.5%)
Epithelioid	10 (9.5%)
Mixoid	9 (8.6%)
Mixed	13 (12.4%)
Surgery	
Primary cytoreduction	73 (69.5%)
Secondary cytoreduction	32 (30.5%)
Adjuvant therapy ^a^	
Chemotherapy	
Yes	89 (84.7%)
No	11 (10.5%)
*n.a.*	5 (4.8%)
Radiotherapy	
Local disease control	12 (63.2%)
Palliative	7 (36.8%)

*n.a.* = not available, ^a^ patients could have received >1 treatment.

**Table 2:** Histology and immunohistochemistry features of uLMS tissue samples.

	uLMS Tissue Samples (N = 112)
Tumor cellularity	10.6 (3–42)
<20%	2 (1.8%)
21–40%	7 (6.2%)
41–60%	4 (3.5%)
61–80%	29 (25.8%)
>80%	70 (62.7%)
Median (range)	90% (10–100)
Tumor necrosis	
<20%	91 (81.2%)
21–40%	14 (12.5%)
41–60%	6 (5.4%)
61–80%	1 (0.9%)
>80%	0
Median (range)	0% (0–70)
Estrogen receptors	
Primary tissue samples (N = 73)	
median (range)	21% (0–100)
Recurrence tissue samples (N = 39)	
median (range)	28% (0–90)
Progesterone receptors	
Primary tissue samples (N = 73)	15% (0–100)
median (range)	
Recurrence tissue samples (N = 39)	18% (0–100)
median (range)	
Ki67	
median (range)	48% (10–90)

**Table 3:** Multivariate analysis of clinical outcomes on FIGO disease stage and genomic alterations.

Variables		PFS		OS	
	N.	HR (CI95%)	*p* Value	HR (CI95%)	*p* Value
FIGO stage					
Advanced/metastatic	19				
Early	54	0.338 (0.161–0.709)	0.004	0.449 (0.211–0.954)	0.037
*TP53* alterations					
Yes	30 *				
No	43	0.510 (0.246–1.056)	0.07	0.312 (0.145–0.670)	0.003
CNA					
Presence	15				
Absent	58	1.102 (0.504–2.410)	0.807	1.056 (0.474–2.353)	0.893
*BRCA* and HRR alterations					
Yes	8 *				
No	65	0.991 (0.419–2.344)	0.983	0.695 (0.293–1.648)	0.409

HR: Hazard ratio; CI95%: 95% Confidence interval. * Only *TP53-* or *BRCA-* and HRR-related alterations; a total of 8 patients carrying both *TP53-* and HRR-related alterations were excluded from the analysis.

## Data Availability

Data available on request from the authors.

## Supplementary Materials

The following supporting information can be downloaded at: https://www.mdpi.com/article/10.3390/cancers14081934/s1; Figure S1. A: PFS and OS in all patients (N = 105); B: PFS and OS in patients with biopsy of tumor samples at primary diagnosis (N = 73); Figure S2: Single Nucleotide Variants (SNVs) and Copy Number Alterations (CNAs) distribution along the analyzed genes; Figure S3: PFS and OS according to patients with CAN presence versus CAN absence; Table S1: Molecular alterations in uLMS, grouped by biopsy site.
