# Supplementary material for: Role of Homologous Recombination Repair (HRR) Genes in Uterine Leiomyosarcomas: A Retrospective Analysis
Source: Cancers (Basel). 2022 Apr 12;14(8):1934. doi: 10.3390/cancers14081934 (PMC9024785; doi:10.3390/cancers14081934)
Supplement: Supplementary file 1 [file cancers-14-01934-s001.zip › cancers-1660929-supplementary.pdf]

cancers

Supplementary Materials: The Role of Homologous Recombination Repair (HRR) Genes in Uterine Leiomyosarcomas: A Retrospective Analysis

Francesca Ciccarone, Matteo Bruno, Elisa De Paolis, Alessia Piermattei, Maria De Bonis, Domenica Lorusso, Gian Franco Zannoni, Nicola Normanno, Angelo Minucci, Giovanni Scambia and Gabriella Ferrandina

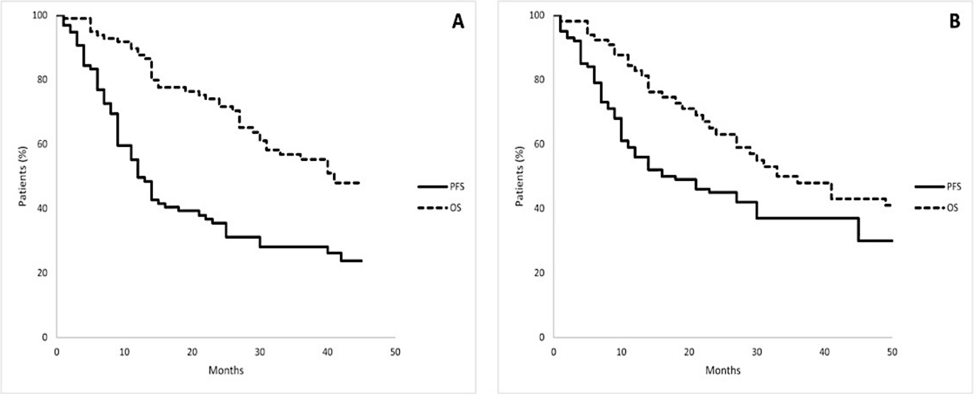

Figure S1. (**A**) PFS and OS in all patients (N = 105); (**B**) PFS and OS in patients with biopsy of tumor samples at primary diagnosis (N = 73);.

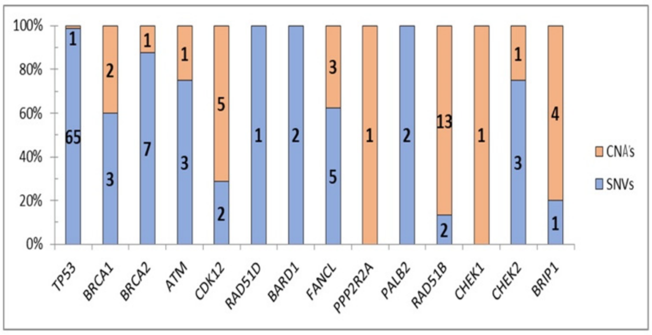

Figure S2. Single Nucleotide Variants (SNVs) and Copy Number Alterations (CNAs) distribution along the analyzed genes.

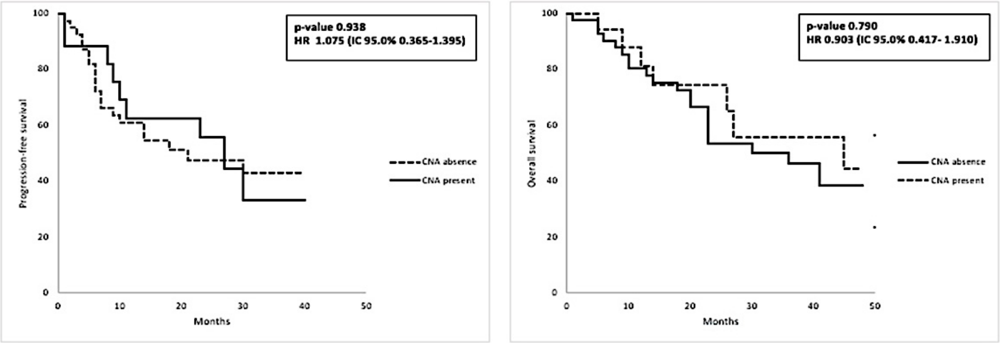

Figure S3. PFS and OS according to patients with CAN presence versus CAN absence.

Table S1. Molecular alterations of uLMS. The table shows the molecular alterations accounted in the uLMS, grouped by biopsy site. uLMS: uterine leyomiosarcomas; rs: reference SNP; VAF%: Variant Allele Frequency %; CNA: Copy Number Alteration**.**

uLMS Group	ID Sample	GENE	cDNA change	Aminoacid change	rs	VAF%	CNA
PRIMARY	uLMS001_P1	TP53	c.524G>A	p.Arg175His	rs28934578	59%	
	uLMS002_P2	TP53	c.843_845del	p.Asp281_Thr284	-	59%	
	uLMS003_P3	TP53	c.994-1G>C	p.?	rs587782272	70%	
	uLMS004_P4	TP53	c.467G>C	p.Arg156Pro	rs371524413	19%	
			c.713G>A	p.Cys238Tyr	rs730882005	60%	
	uLMS005_P5	TP53	c.920-2A>G	p.?	rs397516439	75%	
	uLMS006_P6	TP53	c.843C>G	p.Asp281Glu	rs1057519984	82%	
	uLMS007_P7	TP53	c.786_801delTAATCTACTGG-GACGG	p.Asn263Thr*77	-	48%	
	uLMS008_P8	TP53	c.376-2A>G	p.?	rs786202799	31%	
			c.31G>A	p.Glu11Lys	rs201382018	29%	
	uLMS009_P9	TP53	c.742C>T	p.Arg248Trp	rs121912651	70%	
	uLMS010_P10	TP53	c.731G>A	p.Gly244Asp	rs985033810	42%	
	uLMS011_P11	TP53	c.376T>G	p.Tyr126Asp	rs886039483	56%	
	uLMS012_P12	TP53	c.374C>A	p.Thr125Lys	rs786201057	35%	
	uLMS013_P13	TP53	c.745A>G	p.Arg249Gly	rs587782082	93%	
	uLMS014_P14	TP53	c.569dupC	p.Pro191Serfs*18	-	14%	
	uLMS015_P15	TP53	c.538G>A	p.Glu180Lys	rs879253911	25%	
		TP53	c.714dupT	p.Asn239*	rs1567549651	52%	
	uLMS016_P16	TP53	c.230del	p.Pro77Glnfs*46	-	20%	
			c.428T>C	p.Val143Ala	rs1555526241	21%	
	uLMS017_P17	TP53	c.809T>C	p.Phe270Ser	rs1057519986	48%	
	uLMS018_P18	TP53	c.461G>T	p.Gly154Val	rs762846821	84%	
	uLMS019_P19	TP53	c.478del	p.Met160Trpfs*10	-	37%	
	uLMS020_P20	TP53	c.809T>C	p.Phe270Ser	rs1057519986	27%	RAD51B - Exon 9 to 11 Del
			c.626_627del	p.Arg209Lysfs*6	rs1057517840	20%	
	uLMS021_P21	TP53	c.731G>A	p.Gly244Asp	rs985033810	37%	FANCL - Exon 8 to14 Del
			c.857A>G	p.Glu286Gly	rs1057519985	33%	
	uLMS022_P22	TP53	c.420del	p.Cys141Alafs*29	-	41%	RAD51B - Exon 2 to 7 Ampl
			c.396G>C	p.Lys132Asn	rs866775781	39%	CDK12 - Exon 3 to 8 Ampl
	uLMS023_P23	TP53	c.403_405delinsGG	p.Cys135Glyfs*35	rs1131691036	79%	RAD51B - Exon 11 Ampl
	uLMS024_P24	TP53	c.374C>G	p.Thr125Arg	rs786201057	84%	BRIP1 - Exon 15

							to 20 Ampl
	uLMS025_P25	TP53	c.329G>T	p.Arg110Leu	rs11540654	83%	RAD51B - Exon 10 to 11 Ampl
	uLMS026_P26	TP53	c.1022del	p.Phe341Serfs*4	-	77%	CDK12 - Exon 5 to 8 Ampl
	uLMS027_P27	TP53	c.626_627del	p.Arg209Lysfs*6	rs1057517840	47%	
			c.743G>A	p.Arg248Gln	rs11540652	26%	
		BRCA2	c.4854T>A	p.Asp1618Glu	rs80358708	13%	
	uLMS028_P28	PALB2	c.3451C>T	p.Leu1151Phe	rs786203462	89%	BRCA2 - Exon 2 Del
		TP53	c.646G>C	p.Val216Leu	rs730882025	79%	
	uLMS029_P29	TP53	c.783-4_790del	p.?	-	70%	
		CDK12	c.3814C>G	p.Leu1272Val	-	49%	
	uLMS030_P30	TP53	c.626_627del	p.Arg209Lysfs*6	rs1057517840	78%	
		RAD51D	c.287C>G	p.Ala96Gly	rs1234250425	18%	
	uLMS031_P31	TP53	c.856delGAinsTT	p.Glu286Leu	-	42%	
		TP53	c.818G>A	p.Arg273His	rs28934576	40%	
		BRCA2	c.644_646del	p.Glu215del	rs80359588	85%	
	uLMS032_P32	BRCA1	c.1961dupA	p.Tyr655Valfs*18	rs80357522	73%	
		TP53	c.713G>A	p.Cys238Tyr	rs730882005	69%	
		BRCA2	c.7330G>T	p.Asp2444Tyr	rs398122579	45%	
	uLMS033_P33	TP53	c.457_466delinsGGT	p.Pro153Glyfs*15	-	46%	
		BRCA1	c.981_982del	p.Thr327_Cys328in-sTer	rs80357772	11%	
	uLMS034_P34	BRCA2	c.6591_6592del	p.Glu2198Asnfs*4	rs80359605	58%	
		ATM	c.8773G>A	p.Gly2925Ser	rs876658519	60%	
	uLMS035_P35	RAD51B	c.728A>G	p.Lys243Arg	-	38%	BRCA1 - Exon 10 to 13 Ampl
	uLMS036_P36	CDK12	c.4390C>T	p.Arg1464Trp	-	46%	
	uLMS037_P37	BARD1	c.282_291delinsT	p.Leu95_Ile97del	rs1064795485	39%	
	uLMS038_P38	FANCL	c.1089dupA	p.Cys374Mefs*5	-	8%	
	uLMS039_P39	BRCA2	c.5286T>G	p.Tyr1762Ter	rs80358754	12%	
	uLMS040_P40	CHEK2	c.721+3A>T	p.?	rs587782849	43%	PPP2R2A - Intron 1 to 9 Del
	uLMS041_P41	FANCL	c.238C>G	p.Leu80Val	rs563513081	40%	
METASTATIC DISEASE	uLMS042_R1	TP53	c.560-3T>G	p.?	rs763746485	77%	BRIP1 - Exon 5 to 16 Ampl
	uLMS043_R2	TP53	c.626_627del	p.Arg209Lysfs*6	rs1057517840	60%	
	uLMS044_R3	TP53	c.818G>T	p.Arg273Leu	rs28934576	66%	
	uLMS045_R4	TP53	c.853G>A	p.Glu285Lys	rs112431538	45%	

			c.589G>A	p.Val197Met	rs786204041	43%	
	uLMS046_R5	TP53	c.783-1G>T	p.?	rs1555525367	53%	FANCL - Exon 1 to 7 Del
			c.722C>T	p.Ser241Phe	rs28934573	26%	RAD51B - Exon 8 to 11 Ampl
	uLMS047_R6	TP53	c.522_540dup-CTCATGGTGGGCAGCGC	p.Arg181Alafs*6	-	58%	
	uLMS048_R7	TP53	c.487T>A	p.Tyr163Asn	rs786203436	62%	
	uLMS049_R8	TP53	c.493C>T	p.Gln165*	rs730882001	21%	
	uLMS050_R9	TP53	c.711G>A	p.Met237Ile	rs587782664	33%	RAD51B - Exon 8 to Intron 10 Del
			c.466C>G	p.Arg156Gly	-	32%	
	uLMS051_R10	TP53	c.376-1G>C	p.?	-	89%	
	uLMS052_R11	BRCA1	c.1703C>G	p.Pro568Arg	rs80356910	43%	CHEK1 - Exons 4 to 11 Del; RAD51B - Exons 2 to 5 Del; CDK12 - Exons 2 to 13 Del
		TP53	c.749C>T	p.Pro250Leu	rs1064794311	10%	
	uLMS053_R12	TP53	c.404G>A	p.Cys135Tyr	rs587781991	17%	
		FANCL	c.979C>G	p.Gln327Glu	rs764186411	37%	
	uLMS054_R13	PALB2	c.1368G>T	p.Glu456Asp	rs1555461319	23%	
	uLMS055_R14	FANCL	c.238C>G	p.Leu80Val	rs563513081	41%	
	uLMS056_R15	BARD1	c.1718T>C	p.Ile573Thr	rs587780022	95%	
	uLMS054_R16	BRCA2	c.2078G>A	p.Cys693Tyr	rs771226774	64%	
		BRIP1	c.139C>G	p.Pro47Ala	rs28903098	51%	
		CHEK2	c.475T>C	p.Tyr159His	rs781254437	64%	
	uLMS055_R17	ATM	c.483G>C	p.Gln161His	rs864622742	**5%**	
	uLMS056_R18	ATM	c.2638+3A>G	p.?	rs876660552	**6%**	
	uLMS057_R19	FANCL	c.2T>C	p.Met1?	rs761291501	46%	
		RAD51B	c.728A>G	p.Lys243Arg	rs34594234	46%	
PRIMARY & MATCHED METASTATIC DISEASE	uLMS058_P42	TP53	c.658T>C	p.Thy220His	rs530941076	13%	
	uLMS058_R20	TP53	c.658T>C	p.Thy220His	rs530941076	76%	CDK12 - Exon 1 to 5 Ampl
	uLMS059_P43	-	-	-	-	-	
	uLMS059_R21	TP53	c.377A>G	p.Tyr126Cys	rs1555526335	18%	
	uLMS060_P44	TP53	c.538G>A	p.Glu180Lys	rs879253911	81%	
		CHEK2	c.276dupC	p.Trp93Leufs*15	rs876661156	8%	
	uLMS060_R22	TP53	c.538G>A	p.Glu180Lys	rs879253911	85%	

		TP53	c.981T>G	p.Tyr327*	rs879254077	90%	
		BRCA2	c.2489A>G	p.Asn830Ser	rs786202809	40%	
	uLMS061_P45	TP53	c.818G>A	p.Arg273His	rs28934576	46%	RAD51B - Exon 4 to 7 Ampl; CDK12 - Exon 3 to 8 Ampl
	uLMS061_R23	TP53	c.818G>A	p.Arg273His	rs28934576	84%	
	uLMS062_P46	TP53	c.487T>A	p.(Tyr163Asn)	rs786203436	53%	
	uLMS062_R24	-	-	-	-	-	
	uLMS063_P47	TP53	c.329G>T	p.Arg110Leu	rs11540654	27%	ATM - Exon 23 to 24 Ampl
		TP53	c.830G>A	p.Cys277Tyr	rs763098116	30%	
	uLMS063_R25	-	-	-	-	-	
